# Efficacy and influencing factors of percutaneous sclerotherapy for venous malformations of the knee in children

**DOI:** 10.3389/fped.2026.1910866

**Published:** 2026-07-28

**Authors:** Zhuang Liu, Xin Zhang, Liang Wang, Jing Li, Dan Song

**Affiliations:** Department of Vascular Anomalies and Interventional Radiology, Jinan Children’s Hospital, Jinan, Shandong, China

**Keywords:** children, knee, percutaneous sclerotherapy, synovial involvement, venous malformations

## Abstract

**Objective:**

To evaluate the clinical efficacy, safety, and the factors influencing percutaneous sclerotherapy combined with pingyangmycin and polidocanol foam for treating venous malformations of the knee in children.

**Methods:**

A retrospective analysis was conducted on 13 pediatric patients with knee venous malformations who were treated with percutaneous sclerotherapy under ultrasound combined with digital subtraction angiography guidance between March 2020 and February 2026. Preoperative imaging was performed to classify the lesions as intra-articular, juxta-articular, localized, or diffuse, and to assess synovial involvement. All patients underwent sequential sclerotherapy at intervals of 4–6 weeks. Follow-up outcomes included pain relief assessed using the Visual Analog Scale, lesion volume regression on imaging, and postoperative complications. Subgroup analyses were performed to explore the impact of synovial involvement and lesion morphology on therapeutic response.

**Results:**

The mean age of the 13 patients was 10.5 years, with a mean of 3.2 treatment sessions per patient. Among the 9 patients with preoperative pain, the pain relief rate was 88.9%. The overall imaging efficacy rate (significant + partial response) for lesion volume reduction was 61.5%. The subgroup analysis showed a higher lesion response rate in localized lesions than in diffuse lesions; moreover, patients without synovial involvement achieved better pain relief. All complications were minor transient local reactions of Society of Interventional Radiology grades A–B, with no major complications such as skin necrosis, nerve injury, deep vein thrombosis, or pulmonary embolism.

**Conclusion:**

Percutaneous sclerotherapy with pingyangmycin combined with polidocanol foam is a safe, effective, and minimally invasive method for treating pediatric knee venous malformations, with satisfactory pain relief and lesion regression. Synovial involvement and diffuse lesion morphology are the key adverse prognostic factors. Precise preoperative imaging evaluation and individualized multidisciplinary stratified treatment are essential for optimizing clinical outcomes.

## 1 Introduction

Venous malformations (VMs) are the most common congenital vascular anomalies; they are characterized by dilated, dysplastic venous channels that fail to regress normally (1). These low-flow lesions can occur anywhere in the body, with a considerable proportion involving the extremities, particularly the knee joint (2, 3). Intra-articular or juxta-articular VMs of the knee pose unique clinical challenges, often presenting with chronic pain, recurrent hemarthrosis, swelling, and progressive functional impairment (4, 5). These symptoms arise not only from a mass effect, but recurrent intra-articular bleeding can also induce synovitis, leading to irreversible cartilage damage, premature degenerative joint disease, and severe dysfunction (6, 7).

Management of symptomatic knee VMs requires a multidisciplinary approach. Surgical synovectomy or resection is a keystone treatment that provides pain relief and functional improvement (8, 9), it is not without risks, including intraoperative bleeding, particularly in patients with coagulopathies (10). In recent decades, percutaneous sclerotherapy has emerged as a pivotal, often first-line, minimally invasive treatment modality (11, 12). The procedure involves image-guided injection of sclerosants (e.g., ethanol, sodium tetradecyl sulfate, or polidocanol) to induce endothelial injury, thrombosis, and subsequent fibrosis of malformed venous channels (2).

Despite sclerotherapy being widely adopted, comprehensive evidence specifically evaluating its safety and efficacy for knee VMs remains lacking. Existing data are often extrapolated from studies on VMs at various body sites; this may obscure knee-specific treatment outcomes and risks. Therefore, this article aimed to critically review and synthesize the current evidence on percutaneous sclerotherapy for knee VMs, focusing on its clinical effectiveness, complication spectrum, and the factors influencing treatment success. By doing so, we intend to provide evidence-based insights into optimizing treatment strategies and improving patient outcomes.

## 2 Materials and methods

### 2.1 Study population

This was a retrospective clinical study. Patients diagnosed with and treated for knee VMs at the Jinan Children's Hospital between March 2020 and February 2026 were included in the study. The diagnosis was confirmed based on clinical manifestations and characteristic imaging findings. All patients underwent preoperative magnetic resonance imaging (MRI) or ultrasound (US) to assess the lesion extent and location (intra-articular, juxta-articular, or intramuscular) and specifically to evaluate synovial involvement and venous drainage patterns. The diagnostic criteria were consistent with the ISSVA classification for vascular anomalies (12).

The inclusion criteria were: (1) age <18 years; (2) imaging-confirmed knee VM with or without clinical symptoms (e.g., chronic pain, swelling, recurrent hemarthrosis, or limited mobility); (3) failure or refractoriness to conservative treatment, or the presence of an indication for prophylactic therapy; and (4) receipt of percutaneous sclerotherapy with pingyangmycin and polidocanol foam.

The exclusion criteria were: (1) severe localized intravascular coagulopathy (LIC) that could not be effectively corrected preoperatively, (2) allergy to sclerosants, (3) active infection at the puncture site, and (4) other specific contraindications to sclerotherapy.

This was a retrospective study, and all data collection and analysis strictly adhered to the ethical guidelines of the Declaration of Helsinki. Given the retrospective nature of the study, the ethics committee waived the requirement for written informed consent from individual patients.

### 2.2 Preparation of sclerosants

#### 2.2.1 Preparation of foam sclerosing agent

Using the Tessari technique, 3% polidocanol injection (2 mL: 60 mg/vial, manufactured by Chemische Fabrik Kreussler, Germany) and CO_2_ were mixed in a 1:3 ratio, and rapidly blended into foam via the vortex method. The resulting foam had to be fine and uniform. The intraoperative application dose was 2 mg/kg, with a total dose per treatment ≤100 mg (13, 14).

#### 2.2.2 Preparation of pingyangmycin

Exactly 8 mg of pingyangmycin (8 mg/vial, manufactured by Yanji Company of Jilin Aodong Pharmaceutical Group) was dissolved in 4 mL of contrast medium. The intraoperative dosage was calculated based on a body surface area of 10 mg/m^2^ or 0.3–0.5 mg/kg, with a total dose per treatment ≤8 mg. During treatment, 1–2 mg of dexamethasone was added and mixed.

### 2.3 Treatment protocol

**Figure 1:**
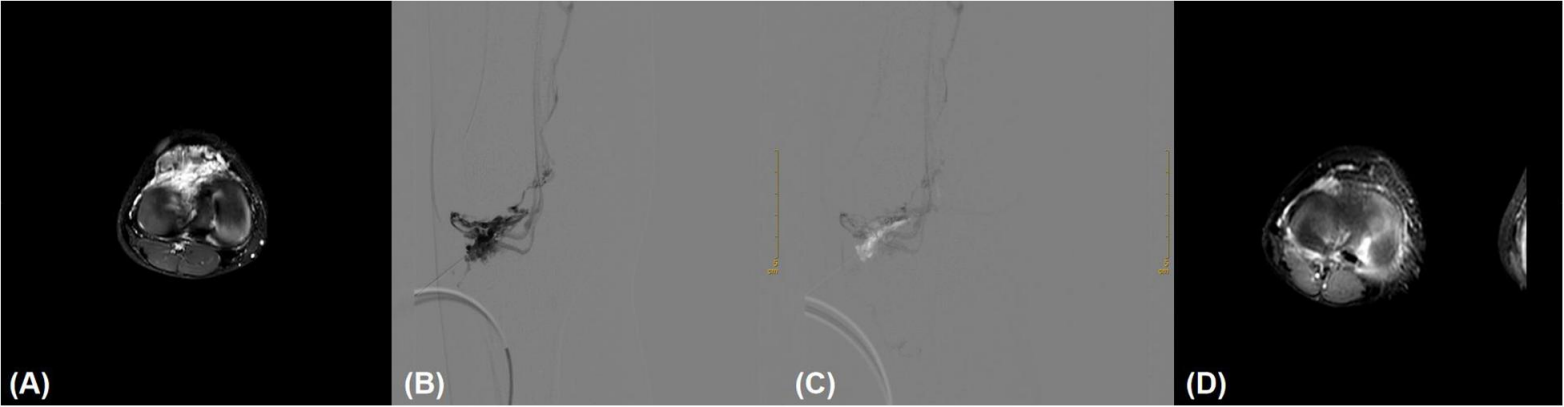
A 9-year-old girl with venous malformation of the right knee who underwent sclerotherapy under DSA guidance. **(A)** Axial fat-suppressed T2-weighted magnetic resonance imaging of the right knee reveals hyperintense venous malformation lesion. **(B)** DSA angiography after local puncture of the lesion revealed the malformed venous mass and draining veins. **(C)** Injection of polidocanol sclerosant. **(D)** Postoperative follow-up MRI showed significant regression of the lesion.

All sclerotherapy procedures were performed under general or local anesthesia. Under the dual guidance of US and digital subtraction angiography (DSA), a 21G puncture needle was percutaneously inserted accurately into the nidus of the VMs while avoiding the adjacent normal neurovascular structures and soft tissues. After proper needle positioning, a mixture of pingyangmycin and contrast medium was injected for intraoperative venography, which clearly delineated the course, drainage pattern, shunt characteristics, and communication between the superficial and deep veins to comprehensively evaluate the hemodynamic features of the lesion (Figure 1).

The injection volume of the sclerosant was individually adjusted according to the lesion size, extent of involvement, and venous outflow velocity. The injection endpoint was defined as complete opacification and filling of the malformed venous cavity, without obvious extravasation or rapid runoff into the deep venous system. All the patients underwent repeated sessions at intervals of 4–6 weeks. The treatment was continued until significant relief of clinical symptoms was achieved or imaging evaluation revealed a maximal therapeutic response with no further potential for lesion regression.

### 2.4 Follow-up and treatment response assessment

All patients were followed up in the outpatient clinic 1–2 months post-procedure, and for those unable to attend in-person visits, follow-up was conducted by physicians via online or telephone contact. Follow-up assessments included physical examinations and imaging evaluations using US or MRI.

Treatment response was evaluated based on symptom improvement and imaging findings as follows:

Symptom assessment: Pain was evaluated using the Visual Analog Scale (VAS).

Imaging assessment: Changes in lesion volume were measured using MRI or US.

Responses were classified thus: (1) complete response, 100% volume reduction with complete symptom resolution; (2) significant response, >85% volume reduction with marked symptom improvement; (3) partial response, 50%–85% volume reduction or partial symptom improvement; and (4) no response, <50% volume reduction or no symptom improvement.

Post-treatment complications were recorded and classified according to the Society of Interventional Radiology (SIR) classification system as minor (transient swelling, local pain, low-grade fever, SIR grade A-B) or major (skin necrosis, nerve injury, deep vein thrombosis, pulmonary embolism, SIR grade C-F).

## 3 Results

### 3.1 Patient demographics and lesion characteristics

During the study period from March 2020 to February 2026, a total of 13 patients (7 male, 6 female) with knee VM underwent treatment. The mean age at treatment was 10.5 years (range, 2–17 years). All patients underwent preoperative MRI or US examination. Based on imaging characteristics, lesions were classified as intra-articular in 5 cases and juxta-articular in 8 cases; among these, synovial involvement was present in 5 cases. Morphologically, there were 7 localized lesions and 6 diffuse lesions.

### 3.2 Treatment details

All patients underwent percutaneous sclerotherapy with pingyangmycin and polidocanol foam (air-to-liquid ratio 1:3 or 1:4). A total of 41 sclerotherapy sessions were performed, with a mean of 3.2 sessions per patient (range: 2–5 sessions).

### 3.3 Treatment outcomes

At the follow-up conducted 1–2 months after the last treatment, the overall clinical and imaging outcomes were assessed as follows:

Symptom improvement: Among the 13 patients, 4 had no pain at initial presentation; of the 9 patients with preoperative pain, 8 (88.9%) achieved pain relief.

Imaging response: Based on MRI or US evaluation, 8 patients (61.5%) achieved a significant or partial response in lesion volume; 5 patients (38.5%) showed no significant volume reduction.

Due to the small sample size and the rarity of venous malformations involving the synovium, no formal statistical comparisons were performed between subgroups. All subgroup differences reported are purely descriptive and should be interpreted with caution.

#### 3.3.1 Subgroup analysis

##### 3.3.1.1 Impact of synovial involvement on treatment outcomes

Patients without synovial involvement had a higher pain relief rate (100%) than those with synovial involvement (80.0%). Of note, among the patients without synovial involvement, 3 had no pain at the initial visit, and among those with synovial involvement, 1 had no pain at the initial visit. The rate of lesion volume reduction (significant or partial response) was 62.5% in patients without synovial involvement and 60.0% in those with synovial involvement. Three patients without synovial involvement showed no significant volume reduction after treatment, which might be attributable to the diffuse lesion morphology or rapid venous outflow.

##### 3.3.1.2 Effect of lesion type on treatment outcomes

Among the patients with localized lesions, 85.7% achieved lesion volume reduction (significant or partial response), whereas 50.0% of those with diffuse lesions achieved volume reduction. The pain relief rate was 85.7% in the localized group and 100.0% in the diffuse group (notably, 3 patients in the localized group and 1 patient in the diffuse group had no pain at presentation).

### 3.4 Complications

All patients experienced varying degrees of postoperative local reactions; however, all complications were minor (SIR grades A-B). The most common complication was transient local swelling (11 cases), which resolved spontaneously within 3–7 days without special management; an additional 2 patients developed local swelling with joint mobility impairment during the treatment course, both of which resolved after conservative therapy. No major complications (SIR grades C-F) such as skin necrosis, permanent nerve injury, deep vein thrombosis, or pulmonary embolism were observed in this study.

## 4 Discussion

Through a retrospective analysis of the clinical data from a cohort of pediatric patients with knee VMs who underwent percutaneous sclerotherapy, we aimed to systematically evaluate the safety and efficacy of this intervention. Our findings are consistent with those of the existing literature and further emphasize the importance of individualized treatment decisions based on lesion characteristics.

However, the unique anatomical environment of the knee, proximity to neurovascular structures, complex synovial recesses, and potential communication with the deep venous system, poses specific challenges that directly impact the safety and efficacy profile of sclerotherapy (15). Moreover, a considerable proportion of patients with extensive VMs present with localized intravascular coagulopathy (LIC), characterized by elevated D-dimer and decreased fibrinogen levels, which in turn complicate perioperative management and increase the risk of bleeding and thromboembolic events (16). Recent evidence indicates that pre-interventional magnetic resonance imaging features such as synovial involvement and signs of osteoarthritis are strong predictors of a poor response to sclerotherapy, underscoring the need for careful patient selection (4).

A major strength of the present study was that all sclerotherapy procedures were performed under real-time combined imaging guidance rather than blind puncture. Intraoperatively, US was first applied to dynamically delineate the extent, depth, and internal echo characteristics of the VMs, enabling accurate targeting of the lesion cavity while avoiding adjacent neurovascular structures. Subsequent DSA venography clearly delineated the angioarchitecture of the malformed veins, including the drainage pathways, shunt patterns, lesion boundaries, and communication between the superficial and deep venous systems.

Real-time US guidance allowed precise visualization of the puncture, sparing the superficial fascia, subcutaneous nerves, and normal superficial venous branches, thereby minimizing iatrogenic injury to the surrounding healthy tissues. DSA fluoroscopic venography further facilitated the optimal injection of sclerosants to fully obliterate abnormal venous channels without extravasation or rapid runoff into the deep venous circulation, which substantially reduced the risk of long-term thromboembolic complications.

Compared with liquid sclerosants, foam agents have superior performance; they displace intraluminal blood to fully fill vessels for controllable, predictable sclerotherapy. Microbubbles increase the contact area with the vascular endothelium, enabling uniform drug distribution, reduced dosage, and minimal toxicity (14, 15).

The combined use of pingyangmycin and polidocanol foam yields prominent synergistic effects: pingyangmycin directly damages vascular endothelial cells, induces chronic local fibrosis, and exerts a sustained remodeling effect on the walls of malformed veins; as a detergent sclerosant, polidocanol foam rapidly disrupts endothelial membrane integrity and promotes venous spasm and luminal obliteration. Their combined application potentiates the therapeutic efficacy, improves the obliteration rate of VMs, and reduces the dosage of each agent (16). This strategy avoids high-dose-related complications, such as skin ulceration, pigmentation, and peripheral nerve injury, making it particularly suitable for pediatric patients with delicate soft tissues and densely distributed vital anatomical structures around the knee joint.

The case data from this study indicate that percutaneous sclerotherapy is generally effective in relieving pain and reducing lesion volume, with a pain relief rate of 88.9% among patients who had preoperative pain symptoms, and an overall lesion volume reduction rate exceeding 60%. This result is largely consistent with the overall response rate (approximately 69%) reported by Gnannt et al. for sclerotherapy of juxta-articular knee VMs (7), confirming the value of this technique as a first-line treatment for symptomatic knee VMs. The small sample size precluded formal statistical testing of subgroup differences. Consequently, all intergroup comparisons should be considered exploratory and descriptive rather than confirmatory. We acknowledge that apparent differences in outcomes between patients with and without synovial involvement may reflect random variation rather than true biological effects.

However, the heterogeneity in treatment efficacy should not be overlooked. Synovial involvement is a critical negative prognostic factor. In this study, patients with clearly documented synovial involvement had lower pain relief rates than those without synovial involvement. This finding is highly consistent with the conclusions of Gnannt et al., who reported that synovial involvement is one of the strongest predictors of a poor response to sclerotherapy (present in 88% of the poor response group) (7). The pathophysiological basis is that lesions invading the synovium not only act as a component of VMs, but are also more prone to inducing recurrent hemarthrosis and chronic synovitis, thereby complicating the source of pain (a mixture of vascular malformations, inflammatory arthropathy, and possible mechanical factors). Therefore, preoperative high-resolution magnetic resonance imaging assessment of synovial status has moved beyond mere diagnostic value to become a crucial prognostic prediction and patient stratification tool (5, 7). For patients with imaging findings suggesting extensive synovial involvement, especially those already showing signs of early joint degeneration, more cautious treatment expectations should be established and a more comprehensive treatment pathway should be considered.

Children are in a critical window of skeletal and joint growth and development. Without early and standardized intervention for knee VMs, recurrent hemarthrosis and chronic synovitis can disturb normal epiphyseal growth, easily leading to lower limb malalignment, joint contracture with limited flexion and extension, and even permanent motor functional deficits. Early implementation of image-guided sclerotherapy can effectively block the progressive advancement of lesions, reduce the frequency of intra-articular hemorrhage, and protect the normal development of the articular cartilage and epiphyses. It possesses irreplaceable clinical significance for avoiding long-term joint disability and improving the long-term quality of life in pediatric patients.

The complex neurovascular anatomy of the knee region places the safety of sclerotherapy on this site under investigation. In this study, all patients reported postprocedural local reactions, but these were limited to “swelling” or “transient mobility impairment,” which are common local inflammatory responses after the procedure; they resolved spontaneously during the follow-up period without leaving permanent functional deficits. Notably, no major complications such as skin necrosis, permanent nerve injury, deep vein thrombosis, or pulmonary embolism occurred in this cohort (2, 11). This relatively favorable safety profile may be related to the type of sclerosant commonly used in this study. The literature clearly indicates that there are significant differences in the risk profiles of different sclerosants. For example, although absolute ethanol is highly potent, its risks of neurotoxicity, tissue necrosis, and deep vein thrombosis/pulmonary embolism are significantly higher than those of detergent sclerosants, such as sodium tetradecyl sulfate and polidocanol (2). The results of this study suggest that, in the knee region, which is dense in nerves and vessels, the choice of sclerosant constitutes an important risk-control strategy. Furthermore, a comprehensive preoperative evaluation, including imaging assessment of venous outflow and the use of techniques such as tourniquets or manual compression when necessary to limit sclerosant entry into the systemic circulation, are essential for preventing systemic complications (11).

Given the limitations of sclerotherapy for diffuse synovium-involving lesions, its role should be considered within a broader multidisciplinary treatment framework. The current consensus on management emphasizes that for knee VMs, especially complex cases, no single optimal method exists and individualized plans should be developed based on lesion size, location, depth, and symptoms (10). For localized, synovium-sparing juxta-articular lesions, image-guided precision sclerotherapy can serve as a curative option; for intra-articular lesions that have invaded the synovium or are diffuse, treatment decisions should be more inclusive and sequential:

(1) Combined surgery: When the lesion predominantly presents with refractory hemarthrosis and mechanical symptoms, surgical excision (often combined with synovectomy) can directly remove the lesion and protect articular cartilage, with adjuvant sclerotherapy for residual or recurrent lesions postoperatively (6); (2) Preoperative adjunct: For large lesions planned for surgical resection, preoperative embolization or sclerotherapy can effectively reduce intraoperative blood loss and create conditions for more complete surgical excision (2); (3) Exploration of drug therapy: For refractory, diffuse lesions unsuitable for surgery or sclerotherapy, targeted drugs (such as the mTOR inhibitor sirolimus) as a novel systemic treatment option have shown potential for stabilizing disease and alleviating symptoms, although their precise role in knee VMs requires further study (11).

### 4.1 Limitations and future directions

This study had several limitations. First, its single-center retrospective design and limited sample size may have introduced a selection bias，selection bias may exist as patients were not randomly assigned to treatment; instead, treatment decisions were made by clinicians based on clinical judgment, which could introduce baseline imbalances in disease severity, lesion characteristics, and patient demographics. To mitigate this, we established strict predefined inclusion and exclusion criteria and restricted the study period to a defined timeframe to ensure consistency in clinical practice patterns. Second, the lack of long-term, uniform imaging follow-up and standardized patient-reported functional outcome measures (e.g., joint-specific functional scoring scales) may have affected the precise assessment of long-term efficacy and functional improvement. Future prospective multicenter studies with more rigorous designs are needed to directly compare the differences in the efficacy and safety of various sclerosants and different injection techniques (e.g., liquid vs. foam) at this specific knee location. Concurrently, further exploration of predictive models based on radiomics or biomarkers to more precisely identify patient subgroups that respond favorably to sclerotherapy alone is important for optimizing clinical decision making.

## 5 Conclusion

In summary, this retrospective study demonstrated that percutaneous sclerotherapy is an effective and minimally invasive intervention for pediatric knee venous that significantly alleviates pain and reduces lesion volume. In this cohort, the technique demonstrated an acceptable safety profile, with mostly minor and transient complications. Synovial involvement is an important anatomical factor predicting poor therapeutic efficacy, and treatment success depends on comprehensive preoperative imaging evaluation (with an emphasis on defining synovial involvement), judicious selection of sclerosants based on lesion characteristics and location to balance efficacy and risk, and, most importantly, integrating sclerotherapy into an individualized, sequential treatment strategy developed by a multidisciplinary team targeting patient symptoms and quality of life.

## Data Availability

The original contributions presented in the study are included in the article/Supplementary Material, further inquiries can be directed to the corresponding author.
